# Influence of Environmental Factors on the Aboveground Biomass of Mature and Postmining Forests in Chocó

**DOI:** 10.3390/life15010098

**Published:** 2025-01-15

**Authors:** David Pérez Abadía, Harley Quinto Mosquera, José María Del Arco

**Affiliations:** 1 Doctorate Program in Conservation and Sustainable Use of Forest Systems, University Institute for Research in Sustainable Forest Management, University of Valladolid, 34004 Palencia, Spain; davidfernandoperez@gmail.com; 2 Agroforestry Engineering Program, Faculty of Engineering, Technological University of Chocó Diego Luis Córdoba, Quibdó 270001, Colombia; 3 Biology Program, Faculty of Natural Sciences, Technological University of Chocó Diego Luis Córdoba, Quibdó 270001, Colombia; 4 Department of Agroforestry Sciences, Institute of Sustainable Forest Management Research UVa_INIA, E.T.S. (Higher Technical School) of Agrarian Engineering of Palencia, University of Valladolid, 34004 Palencia, Spain; josemaria.arco@uva.es

**Keywords:** biodiversity, carbon, global climate change, secondary forests, succession

## Abstract

Environmental factors control the accumulation of aboveground biomass (AB) in tropical forests, along with the role of AB in climate change mitigation. As such, the objective of this study was to evaluate the influence of factors such as forest type, succession, abundance of individuals, species richness, height, diameter, texture, and soil nutrient levels on the AB in mature and postmining forests in Chocó, Colombia. Five plots each were set up in primary and postmining forests with 15 and 30 years of regeneration, in which the amount of AB was measured and related to the environmental factors. The AB was 178.32 t ha^−1^ in the mature forests and 35.17 and 56.3 t ha^−1^ after 15 and 30 years of postmining regeneration, respectively. Furthermore, the general AB level was determined by the ecosystem type, diameter, richness, abundance, Shannon evenness, and Margalef diversity. In mature forests, the AB amount was positively influenced by height and richness, and negatively influenced by dominance and evenness; in areas degraded by mining, the AB level was positively related to richness and equity, and negatively related to species diversity and soil silt. In summary, environmental factors determine the carbon storage in the forests in Chocó. Mining reduces the function of these ecosystems in mitigating climate change.

## 1. Introduction

Tropical forests are considered the most important terrestrial ecosystems on the planet due to their geographical extent, ecological complexity, high biodiversity, and rates of endemism [1,2]. In addition, tropical forests play a fundamental role in the global carbon balance and the mitigation of global warming, due to their high rates of atmospheric carbon capture and storage [2,3]. Pan et al. [2] reported that primary forests have the highest tree biomass (aboveground + belowground), with an average of 327.8 tons per hectare (t ha^−1^) [2]. Additionally, the aboveground biomass (AB) amount in tropical rainforests was found to be between 174.23 and 541.6 t ha^−1^ [4]. Thus, the relevance of these terrestrial ecosystems in terms of mitigating global climate change has been verified [3]; however, the amount of AB in different tropical regions varies widely.

The large variation in the AB recorded in tropical forests is largely due to its complexity and ability to be influenced by climatic (precipitation, temperature, and humidity), biological (species composition, type of species, and ecological group), and environmental (age of the substrate, soil fertility, topography, and altitude) factors. Furthermore, the accumulation of AB involves processes such as plant establishment, growth, mortality, succession, and disturbances [5,6,7]. Asner et al. [5] observed that the amount of AB is determined by soil drainage, precipitation, temperature (altitude), dominant species composition, and substrate age in tropical rainforests in Hawaii [5]. Oberleitner et al. [8] reported that the amount of AB is related to the age of the substrate, forest cover, and species richness in forests in Costa Rica. These studies demonstrate the importance of environmental and biological factors in the accumulation of AB in tropical forests.

Poorter et al. [6] found that the AB of trees is strongly affected by precipitation, average tree diameter, and species diversity through an analysis at the neotropical forest scale; soil fertility was weakly related to biomass [6]. For this reason, and based on the results of Asner et al. [5], Poorter et al. [6], and Oberleitner et al. [8], we expected the highest amounts of AB and carbon accumulation to be found in tropical forests with high levels of rainfall and species diversity. However, we need to understand the processes that occur in tropical forests with high rainfall in the relationship between these environmental factors and AB accumulation. To answer this question, we evaluated the AB accumulation in forests with high rainfall and biodiversity. Although Poorter et al. [6] studied 59 sites and 2050 permanent research plots, the range of precipitation in the area was between 750 and 4350 mm annually. Therefore, how environmental and biological factors influence the AB in tropical forests with higher rainfall is unknown. The biogeographical region of Chocó provides a suitable context in which to study the influence of these factors on AB under excessive rainfall conditions. Chocó is characterized by high biodiversity, lowland forest ecosystems, and one of the highest rainfall totals in the world (≈10,000 mm annually) [1,9], providing a suitable site to study the relationship between environmental factors and AB accumulation under high rainfall amounts.

In the forests of Chocó, the stocks and flows of biomass and carbon have been quantified. Faber-Langendoen and Gentry [10] estimated the AB in the forest ecosystems of Tutunendo and Bajo Calima as being between 178.1 and 210.9 t ha^−1^ on acidic soils that were poor in nutrients and rich in organic matter [10]. Three decades later, AB values of between 156.8 and 217.85 t ha^−1^ were recorded in the tropical rainforests in the towns of Opogodó and Pacurita in the region, with AB accumulation rates of 7.8 and 10.9 t ha^−1^ year^−1^, respectively [11]. However, the influence of species richness, forest type, soil nutrients, and variation in precipitation on the AB in the region (which has one of the highest annual rainfall amounts in the world) was unknown.

The Chocó forests, with their high biodiversity and endemism [1], play an important role in mitigating global climate change, having a forest area of ≈7.8 million hectares in Colombia, of which 80% are well-preserved and mature forests. However, those in the local communities constantly carry out open-pit mining for gold and platinum in these ecosystems, which destroys and degrades approximately 360 hectares of forest each year [12]. These activities considerably reduce the AB in these ecosystems and the ability of these ecosystems to contribute to the mitigation of global climate change because, after mining, the ecosystem requires more than 300 years to recover [13]. As such, in this study, we aimed to answer the following question: how do environmental and biological factors such as forest type (primary and secondary), succession time, abundance, tree species dominance, species richness, diameter, texture, and soil drainage affect the variation in AB amount in mature and postmining forests in Chocó, an area with one of the highest rainfall amounts in the world?

## 2. Materials and Methods

### 2.1. Study Area

**Figure 1:**
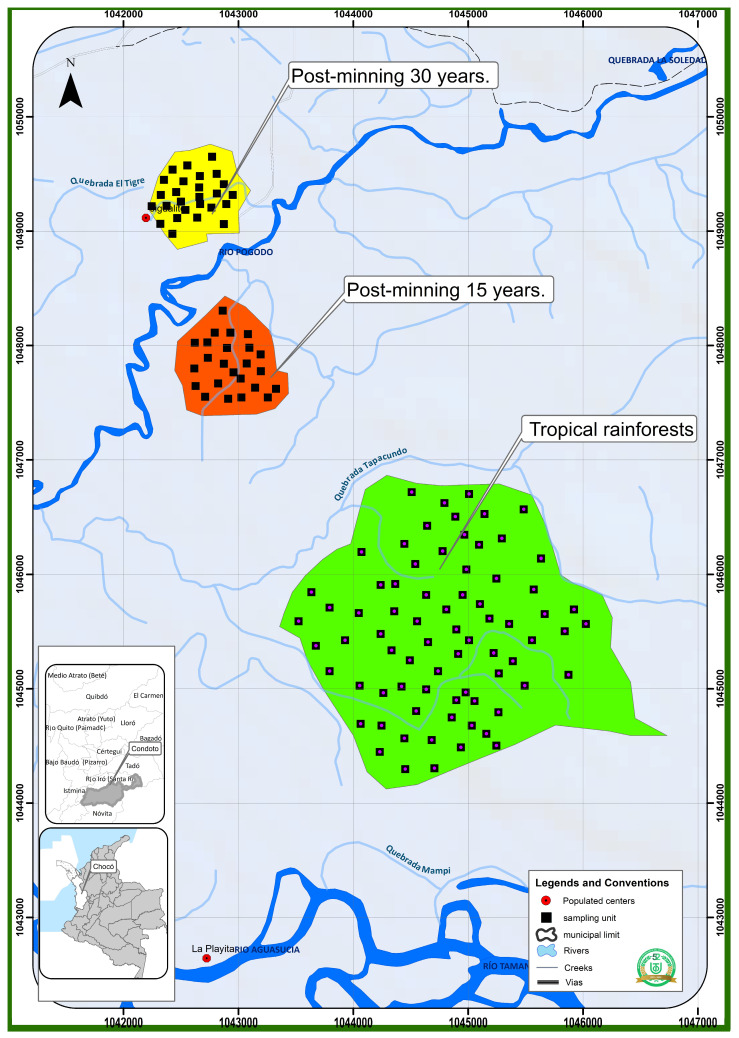
Study area, Chocó, Colombia.

This study was conducted in different forests of Chocó, Colombia, specifically in the tropical rainforests of the towns of Pacurita (5°41′55″ N, 76°35′59″ W) and Opogodó (5°04′07″ N, 76°64′74″ W), as well as in the secondary forests of Jigualito (5°06′01″ N, 76°32′44″ W) (Figure 1). These forests were in different stages of succession due to open-pit mining activity. These areas are part of the north central subregion of Chocó, which includes the upper basins of the Atrato and San Juan Rivers and features foothill and low hill landscapes, humid terrace soils, and transitional sedimentary rock [9]. The annual rainfall and altitude of the Opogodó forests are 8000 mm and 80 m, respectively, and the topography is mostly flat. The annual precipitation and altitude in Pacurita are 10,000 mm and 130 m, respectively, and the topography is broken [11]. In Jigualito, the annual precipitation is 8000 mm, the altitude is 70 m and the topography flat. The forests in this locality are mostly secondary, determined by the time when open-pit mining activities were halted. Consequently, the forests are in various stages of recovery after open-pit gold and platinum mining activities were conducted at different times, which have led to the formation of forests in different successional stages [13]. A map of the study area is provided.

The soils of the four forests studied were Ultisols that differed in their nutrient contents and texture. Specifically, the soils were extremely acidic, with high aluminum saturation in the Salero and Pacurita forests. The soils of the Opogodó forests had high concentrations of OM and total N. The edaphic P, Mg, Ca, and CICE values were low, those of K were intermediate [11]. The soils of Jigualito, an area in which mining activities were previously carried out, were characterized by large amounts of rocky material and sand. The soils in this area were acidic, with high contents of OM, P, and total N, as well as intermediate concentrations of Mg and K. However, the calcium content was low, and the aluminum content was high (Table 1).

The composition of the tree layer in Opogodó was dominated by species such as Wettinia quinaria, Mabea occidentalis, Calophyllum auratum, Eschweilera sclerophylla, and Oenocarpus bataua. The Pacurita tree layer was dominated by Calophyllum auratum, Eschweilera sclerophylla, Oenocarpus bataua, Protium apiculatum, and Brosimum utile. Opogodó was dominated by botanical families such as the Arecaceae, Fabaceae, Lecythidaceae, Hypericaceae, Sapotaceae, and Euphorbiaceae. The dominant families in Pacurita were the Arecaceae, Sapotaceae, Lecythidaceae, Clusiaceae, Moraceae, and Chrysobalanaceae [14]. Between 75 and 90 tree species per hectare have been recorded in the forests of Opogodó; an average of 95 species per hectare has been recorded in Pacurita. The dominant species in the forests of Jigualito were Anthurium formosum, Anthurium alatum, Philodendron acutatum, Cespedesia spathulata, Croton chocoanus, Glossoloma, and Tonina fluviatilis, with botanical families such as the Annonaceae, Areaceae, Asteraceae Buseraceae, Chrysobalanaceae, Clusiaceae, Cyatheaceae, and Melastomataceae. Table 1 summarizes the structural, ecological, and edaphic characteristics of the primary and recovering forests (abandoned mines) evaluated in this study.

### 2.2. Plot Establishment

Five permanent 1 ha plots were established, divided into 25 quadrats measuring 20 × 20 m (400 m^2^), which we used as the sampling units in the primary forests of Opogodó and Pacurita. Five plots measuring 25 × 25 m (625 m^2^) were established in the areas degraded by open-pit gold and platinum mines in Jigualito that had been abandoned for more than 15 years, which were subdivided into 25 quadrats measuring 5 × 5 m (25 m^2^). Similarly, five permanent plots measuring 50 × 50 m (2500 m^2^) were installed in another forest area that was degraded by mines, which had been regenerating for more than 30 years. Within each sampling unit, 25 quadrats measuring 10 × 10 m (100 m^2^) were established. Each of the quadrats in the plots was used as a sampling unit for measurements of soil, diversity, structure, and AB of the trees. For comparisons between sampling units, the variables were evaluated at the quadrat and hectare levels.

### 2.3. Measurement of Tree Diameter and Height

The diameter at breast height (DBH) (1.30 m above ground level) was measured for all trees with DBH ≥ 10 cm in the established plots. These measurements were recorded from the cylindrical part of the tree in areas free of nodes, branches, buds, and/or adventitious roots between the months of August and October 2020 in the Jigualito, Opogodó, and Pacurita forests. The growth habit of the measured trees was categorized as tree, vine, liana, and palm; the particular characteristics and observations of each individual were noted. The perimeter of the tree trunk where the DBH was measured was marked with yellow asphalt paint to guarantee that all subsequent measurements were recorded in the same area as the first measurement. In addition, aluminum plates were placed on the grid and plot. The tree height was determined with a Sutton Clinometer at fixed distances of 10 m from the individuals. The diameter and height data of the trees were averaged at the sampling unit or quadrate level.

### 2.4. Taxonomic Identification and Classification into Functional Groups

All morphospecies were identified up to the highest possible taxonomic level (indeterminate NN, species, genus, and family) using specialized keys and via comparison with the material deposited in the collection of the CHOCO herbarium of the Technological University of Chocó. Species richness and the abundance of individuals, as well as the Simpson, Shannon diversity, Margalef, and Berger–Parker indices were determined using the taxonomic data from the permanent plots [14]. These diversity indices were calculated at the sampling unit or quadrat level for comparison on the same scale as the other environmental and biological variables.

### 2.5. Estimation and Classification of Wood Density

The values published in two international databases of wood density generated in tropical forests were used to estimate and classify wood density [15]. The average of the genus or family was used for cases where a species or genus in the plots was not reported in these databases. The average of the plot was used for taxonomically indeterminate individuals.

### 2.6. Soil Analysis

Composite soil samples were obtained at a depth of 20 cm with a cylindrical borehole in each of the sampling units (quadrats). The soil samples were sent to the Biogeochemistry Laboratory of the National University of Colombia, Medellín headquarters, where the physicochemical parameters of the soil were determined using the following techniques: texture with the Bouyoucos method; pH with a soil potentiometer; water/organic matter (OM) 1:2 with the Walkley and Black method and volumetrics; nitrogen with the Micro-Kjeldahl method; phosphorus with L ascorbic acid and UV–Vis spectrophotometry; and Ca, Mg, and K with 1 N ammonium acetate and the neutral and atomic absorption method [11].

### 2.7. Forest AB Estimation

Allometric equations designed for humid and tropical rain forests were used to determine the AB in the forests. The equation proposed by Álvarez et al. [16] was used because it was generated using data on the wood density, diameter, and height of trees in the Colombian Pacific region:

AB = exp (−2.857 + 2.081 × ln DBH + 0.587 × ln TH+ 0.453 × ln Dw)

where *AB* is the aboveground biomass of the trees in kilograms, *DBH* is the diameter at breast height of the trees, TH is the total height of the trees, *Dw* is the density of the wood, and ln is the natural logarithm.

### 2.8. General Data Analysis

Normality was initially evaluated with the Shapiro–Wilk and kurtosis tests to evaluate the effects of the following factors of AB: forest type; succession time; abundance of individuals; average tree diameter; average tree height, species richness; Shannon diversity, Margalef, and Berger–Parker indices; acidity; organic matter, total nitrogen, phosphorus, aluminum, calcium, magnesium, and potassium contents; effective cation exchange capacity (ECEC); and percentages of sand, clay, and silt. Then, nonparametric Mann–Whitney, Kruskal–Wallis (Kw), and Duncan’s multiple range tests were used to evaluate the variation in the AB of the forests, depending on the type of forest (primary or postmining forest), succession duration (15, 30, or 300 years), and location, because the data did not meet the assumptions (normality and homogeneity of variances) for parametric tests. The tree height and diameter values were determined for each individual, whereas the variables used in the analyses of average DBH and average tree height were determined at the quadrat and plot levels in the estimation of BA.

Principal component analysis (PCA) and general linear models (GLMs) were used to determine the influence of the variables on the aboveground biomass of the trees. Multiple regression was performed with a selection of the significant variables using the backward method. This analysis was conducted at the general level (with all ecosystems) for the primary forests and successional forests (previously degraded by mining) and at the sampling unit (quadrat) level. Diversity analyses (Simpson, Shannon, Margalef, and Berger–Parker indices), as well as Mann–Whitney, Kruskal–Wallis, Duncan’s, MLG, and multiple regression tests, were performed in the R programming environment [17].

## 3. Results

The AB of trees in the primary forests in Chocó presented an average (±standard error) value of 178.32 ± 13.2 t ha^−1^, while, in the recovering forests that were previously affected by open-pit mining, the AB was 45.7 ± 3.5 t ha^−1^, with statistically significant differences between these forest types (Mann–Whitney = 9.405; *p*-value < 0.0001). The average AB values were 35.17 ± 5.6 and 56.3 ± 3.05 t ha^−1^ in the forests after 15 and 30 years of succession after mining, respectively. The AB did not significantly differ among the areas previously degraded by mining, but the average AB significantly differed from that in the primary forests (Kruskal–Wallis = 90.25; *p*-value < 0.0001). Significant differences were noted in the AB between locations (Kruskal–Wallis = 94.38; *p*-value < 0.0001). We observed that the average AB was highest in the Pacurita forests, at 214.82 t ha^−1^, and lowest in the recovering forests of Jigualito, at 45.73 t ha^−1^ (Table 2). In summary, the AB amount differed depending on the type of forest, succession duration, and locations.

**Figure 2:**
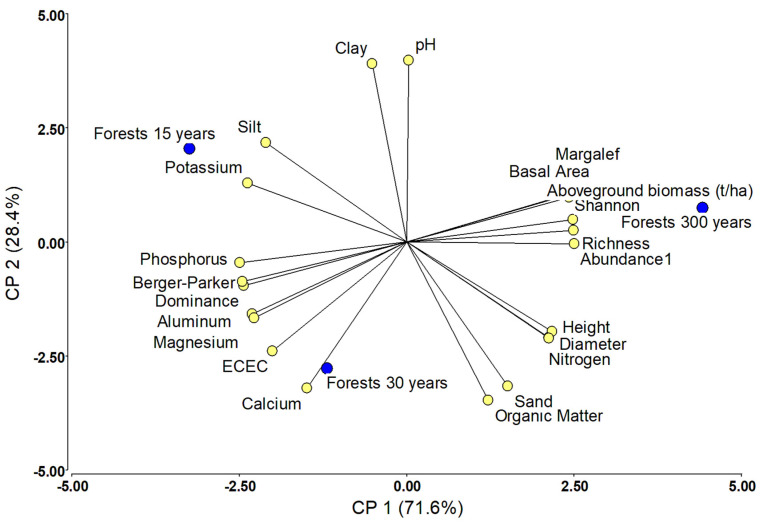
Principal component analysis (PCA) of ecological variables (biomass, structure, richness, and diversity) and edaphic variables (nutrients and texture) of primary and postmining forests in biogeographical region of Chocó.

Regarding the environmental, structural, and ecological factors affecting the AB in all studied forests, AB was significantly determined by the type of ecosystem, average diameter, species richness, abundance of individuals, and the Shannon and Margalef indices (F test = 12.94; *p*-value < 0.001). In this analysis, the variables explained 38.1% of the variation in the AB (Table 3). In primary forests only (Pacurita and Opogodó), the AB was significantly positively influenced by the average tree height and species richness, and negatively influenced by dominance and Shannon’s equity (F test = 24.45; *p*-value < 0.001). In this analysis, the variables explained 44.49% of the variation in the AB (Table 4). Finally, in areas previously degraded by mining, AB was significantly related to species richness and Shannon’s diversity index, and negatively related to the Margalef index and the percentage of soil silt (F test = 10.77; *p*-value < 0.001). In this analysis, the variables explained 54.2% of the variation in the AB (Table 5). The results of the general linear model (GLM) showed that the interaction of variables such as successional stage*nitrogen (F test = 4.27; *p*-value = 0.0411), successional stage*organic matter (F test = 5.97; *p*-value = 0.0161), successional stage*phosphorus (F test = 3.48; *p*-value = 0.064), successional stage*organic matter*richness (F test = 4.65; *p*-value = 0.033), and successional stage*dominance (F test = 5.21; *p*-value = 0.024) significantly influenced aboveground biomass (Table S1 in the Supplementary Materials). The PCA results showed that the AB, Margalef index, Shannon index, species richness, basal area, abundance of individuals, tree height, and tree diameter, as well as nitrogen, organic matter, and sand contents were collinearly related (Figure 2). The PCA results showed that the Simpson and Berger–Parker indices, as well as phosphorus, potassium, aluminum, magnesium, calcium, and silt contents, were collinearly related, especially in forests degraded by mining (Figure 2). PC1 and PC2 explained 71.6% and 28.4% of the variation, respectively (Figure 2).

## 4. Discussion

### 4.1. Aboveground Biomass of Trees in Primary Forests and Forests Degraded by Mining in Chocó

The aboveground biomass of the trees (178.32 t ha^−1^) in the primary forests of Chocó is within the range of 174.23 to 541.6 t ha^−1^ reported for tropical rainforests [4], although the values are close to the lower limit. Similarly, the aboveground biomass values in Chocó are within the range of 98.2 to 295.1 t ha^−1^ reported for different types of tropical forest, but are below the average (243.8 t ha^−1^) reported for Colombia [18]. However, the values for the tropical rainforests in Chocó are similar to the average reported by Phillips et al. [18] of 172.2 t ha^−1^ for this same type of forest. Additionally, the aboveground biomass recorded in this study is similar to that observed in other mature forests in the region. For example, Faber-Langendoen and Gentry [10] estimated an aboveground biomass of between 178.1 and 210.9 t ha^−1^ in the forests of Tutunendo and Bajo Calima. In general, the results show that the aboveground biomass in the Chocó rainforests has intermediate values compared with other types of tropical rainforest.

In the secondary forests that were previously affected by open-pit mining in Chocó, with successional ages of 15 and 30 years, the average aboveground biomass was 35.17 and 56.3 t ha^−1^ (45, 7 t ha^−1^ average), respectively, which is within the range of 20 to 225 t ha^−1^ (average of 121.8 t ha^−1^) reported for secondary tropical forests after 20 years of succession [7]. The aboveground biomass is 27% of that of a mature forest at this successional age; this value would reach 90% after 66 years [7]. The AB in secondary forests was recorded as being up to 52% of that of a primary forest after 20 years of recovery [8].

In this study, the AB in the forests 20 years after mine closure was as high as 40.65 t ha^−1^, corresponding to 22.8% of the aboveground biomass of a mature forest in the region. After 30 years of succession, 31% of the aboveground biomass of a primary forest was reached in the region. That is, the aboveground biomass of a primary forest will be reached in 95 years if the rate of carbon accumulation in the ecosystem degraded by mining remains constant. However, previous modeling showed that these ecosystems can take more than 300 years to reach an aboveground biomass similar to that of the primary forests in the region [13]. This shows that in areas degraded by mining, the accumulation of aboveground biomass tends to be slower than that in secondary forests that have been affected by other types of disturbances, such as agriculture, logging, and/or livestock [7,19].

We found that the areas degraded by mining captured less carbon by comparing the aboveground biomass accumulation rate of 3.05 t ha^−1^ year^−1^ in tropical secondary forests after 20 years of succession after use for agriculture and livestock [7], with those recorded in forests degraded by mining in Chocó of 2.34 and 1.87 t ha^−1^ year^−1^ in forests after 15 and 30 years of succession, respectively. This finding aligns with those reported by Quinto et al. [13] in other localities in Chocó, and Kalamandeen et al. [19] for Amazon mining areas. Furthermore, the reduced carbon capture rate of this type of ecosystem is corroborated by the tropical rainforests in the region accumulating aboveground biomass at 7.8 to 10.9 t ha^−1^ year^−1^ [11]. In summary, mining substantially affected the accumulation of carbon in the ecosystem, weakening the role of the ecosystem in mitigating the effects of climate change.

### 4.2. Influence of Environmental Factors on Aboveground Biomass in Forests and Areas Degraded by Mining in Chocó

The differences in the aboveground biomass depended on the type of forest, succession time, and location in the Chocó forests, similar to what was reported by Poorter et al. [7], Kalamandeen et al. [19], and Oberleitner et al. [8]. The aboveground biomass was determined by the type of ecosystem, average tree diameter, species richness, abundance of individuals, Shannon’s equity, and Margalef diversity at the general level. These results are similar to those reported by Poorter et al. [6], who found that the amount of aboveground biomass was strongly dependent on precipitation, average tree diameter, and species diversity in an analysis at the neotropical forest scale; aboveground biomass was weakly related to soil [6]. Additionally, Chisholm et al. [20] observed a positive relationship between aboveground biomass and tree species diversity. A greater species richness likely increases the variation in the characteristics of the species present in a community, which results in niche complementarity, high resource capture, increased efficiency in resource use, high productivity [6], and, consequently, a greater aboveground tree biomass. In summary, tropical forest ecosystems that have large trees (diameter) and with greater species diversity tend to accumulate more carbon.

Aboveground biomass was previously found to be directly related to the structural characteristics of the forest, such as the basal area, abundance of individuals, tree height, tree crown size, wood density, and abundance of large trees [6]. For example, in a study carried out on pantropical forests, 70% of the variation in aboveground biomass was found to be due to the abundance of large trees (DBH > 70 cm) [21], showing the importance of not only the abundance of individuals but also their sizes. These results align with our observations because aboveground biomass was significantly related to structural variables such as average tree diameter and the abundance of individuals. Consequently, tree diameter in particular appears to be related to the aboveground biomass because of being one of the variables used in the allometric determination, as well as the tree height and density of the wood, of aboveground biomass [16]. Consequently, tree diameter is one of the variables that best explains aboveground biomass [6]. Another important aspect to consider is that the region’s forests receive high amounts of precipitation (8000 mm annually); the modeling of Poorter et al. [6] estimated a decrease in aboveground biomass within this context. This would explain the relatively low levels of aboveground biomass (178.32 t ha^−1^) recorded in the primary forests in the region.

High levels of precipitation produce a lower aboveground tree biomass [6], because rain increases cloudiness and reduces solar radiation. In addition, excessive precipitation tends to decrease the net primary aboveground productivity of tropical forests [22], thereby reducing the aboveground tree biomass. According to Austin and Vitousek [23], high precipitation levels reduce the amount of nutrients and soil fertility, which reduces the carbon capture rate of ecosystems, because productivity is positively related with soil fertility [24,25,26]. For example, Paoli et al. [25] found that carbon sequestration in tropical forest ecosystems increases with soil nutrient availability. Aragão et al. [24] reported that the carbon sequestration (total and fine roots) of the tropical forests in the Amazon increases with soil P content. Similarly, Cleveland et al. [26] reported that P availability positively influences the carbon sequestration rates in tropical humid forests. These findings could explain the relatively low aboveground biomass in some of the Chocó forests, as the soils have low nutrient contents. Although the physicochemical characteristics of the soil were not significantly related to aboveground biomass, as evidenced in other tropical forests [6], the weak relationship with edaphic conditions occurred because many of the tree species present in these forests are adapted to nutrient-poor soils, and spatial changes in their content and edaphic availability do not produce strong variations in aboveground biomass.

In areas previously degraded by mining, AB was found to be positively related to species richness and Shannon evenness, and negatively related to Margalef diversity and the soil’s silt content. These findings are similar to those reported by Oberleitner et al. [8], who found that aboveground biomass is positively related to the age of the substrate and forest cover, and negatively related to species richness in secondary forests in Costa Rica. Asner et al. [5] observed that aboveground biomass amounts are positively affected by poor soil drainage, precipitation, altitude, the dominant species composition, and substrate age in tropical rainforests in Hawaii [5]. The dominance of some tree species (*Philodendron acutatum*, *Cespedesia spathulata*, and *Croton chocoanus*) adapted to recovering areas leads to the aboveground biomass amount being inversely proportional to species richness, especially in areas with high toxic aluminum contents, where most species do not properly develop. This is likely to occur in the areas degraded by mining considered in this study, as the soils have high aluminum contents.

In forested areas degraded by mining, the aboveground biomass was not only related to tree diversity, but also to the soil’s silt content. This result aligns with that reported by Kalamandeen et al. [19], who stated that the recovery of biomass in areas deforested by mining is strongly conditioned by soil nutrient contents, especially nitrogen. Poorter et al. [7] observed that the recovery of aboveground biomass in secondary forests is related to water availability, forest cover, previous land use, and soil fertility in terms of cation exchange capacity (CEC), which shows the importance of the soil in the recovery of aboveground biomass in ecosystems previously degraded by mining. Furthermore, in mining areas, soil is generally removed, and a new substrate is generated that needs to be colonized, which is why the AB produced during primary succession [13,19] requires longer to reach that of a primary forest. All of the above results show the importance of environmental, structural, and biological factors in the accumulation of aboveground biomass and carbon in primary tropical forests and in succession (recovery) processes.

In conclusion, the carbon capture and storage in the Chocó forests is directly related to factors such as species diversity, tree size, soil, forest type, successional age, and previous use of the ecosystem, among others. As such, biodiversity conservation programs should be developed through initiatives such as the reduction in greenhouse gas emissions from deforestation and forest degradation (REDD+) projects [3], which provide comprehensive co-benefit strategies in which species and ecosystems are conserved to substantially contribute to the mitigation of the effects of climate change, as well as to the sustainable development of rural communities in the region.

## Figures and Tables

**Table 1:** Structural, ecological, and edaphic characteristics of the primary and recovering (from abandoned mines) forests evaluated in Chocó.

Location	Pacurita	Opogodó	Jigualito	
Ecosystem Type	Primary Forests		Abandoned Mine	
Succession Time (Years)	≈300		15	30
Average tree diameter in quadrat	15.99	16.86	5.57	13.89
Average tree height in quadrat	15.44	15.83	7.46	13.44
Richness	22.96	19.11	5.56	8.68
Abundance	37.82	30.64	11.12	17.36
Simpson diversity	0.06	0.08	0.31	0.31
Shannon	2.97	2.77	1.42	1.44
Margalef	6.07	5.31	1.75	1.73
Berger–Parker index	0.13	0.15	0.44	0.43
pH	4.03	4.98	4.64	4.47
Organic matter (%)	4.07	11.94	5.07	10.26
Phosphorus (ppm)	1.37	1.33	30.73	26.16
Total nitrogen (%)	0.20	0.61	0.23	0.40
Aluminum (cmol/kg)	0.94	0.13	2.84	3.29
Calcium (cmol/kg)	0.35	0.39	1.28	2.85
Magnesium (cmol/kg)	0.18	0.28	1.35	1.60
Potassium (cmol/kg)	0.17	0.23	0.51	0.34
ECEC (cmol/kg)	1.65	1.03	5.71	8.40
Sand (%)	53.36	85.72	66.08	73.41
Silt (%)	28.12	13.24	23.84	20.35
Clay (%)	18.52	1.04	10.08	6.06

**Table 2:** Aboveground biomass (t ha^−1^) of trees according to forest type, succession duration (years), and location. The aboveground biomass values are reported as the average ± standard error; the letters *a*, *b*, and *c* indicate significant differences between the aboveground biomass averages. The asterisks (*) indicate the significance of the test: ***, *p*-value < 0.001; ND, not significant.

Forest Type	Aboveground Biomass	Mann–Whitney Test
Primary forests	178.32 ± 13.29 *a*	9.405 ***
Successional forests (mines)	45.73 ± 3.5 *b*	
**Succession Duration (years)**	**Aboveground Biomass**	**Kruskal–Wallis Test**
15 years (abandoned mines)	35.17 ± 5.6 *b*	90.25 ***
30 years (abandoned mines)	56.3 ± 3.05 *b*	
300 years (forest)	178.32 ± 13.29 *a*	
**Location**	**Aboveground Biomass**	**Kruskal–Wallis Test**
Pacurita (forest)	214.82 ± 26.98 *a*	94.38 ***
Opogodó (forest)	153.98 ± 12.33 *b*	
Jigualito (abandoned mines)	45.73 ± 3.5 *c*	

**Table 3:** Analysis of variance for aboveground biomass of trees as a function of structural and ecological variables for primary forests and abandoned mines of Chocó biogeographic region. Where R^2^ = 41.3%, R^2^ (adjusted) = 38.1%.

	Sum of Squares	Gf	Middle Square	F Test	*p*-Value
Model	1.40584 × 10^6^	9	156,205	12.94	0.0000
Residue	1.9915 × 10^6^	165	12,069.7		
Total (Corr.)	3.39735 × 10^6^	174			
	**Sum of Squares**	**Gf**	**Middle Square**	**F Test**	***p*-Value**
Ecosystem type	37,957.9	1	37,957.9	3.14	0.0780
Average diameter	85,029.3	1	85,029.3	7.04	0.0087
Average height	2585.05	1	2585.05	0.21	0.6441
Richness	73,722.9	1	73,722.9	6.11	0.0145
Abundance	17,289.0	1	17,289.0	14.32	0.0002
Dominance Simpson	8010.23	1	8010.23	0.66	0.4164
Shannon	47,677.5	1	47,677.5	3.95	0.0485
Margalef	18,044.3	1	18,044.3	14.95	0.0002
Berger–Parker	427.072	1	427.072	0.04	0.8510
Residue	1.9915 × 10^6^	165	12,069.7		
Total (Corr.)	3.39735 × 10^6^	174			

**Table 4:** Analysis of variance and multiple regression for aboveground tree biomass as a function of structural and ecological variables for primary forests in Chocó. R^2^ = 46.39%, R^2^ (adjusted) = 44.49%. The *backward* method was used to select significant variables.

	Sum of Squares	Gf	Middle Square	F Test	*p*-Value
Model	1.22119 × 10^6^	4	30,529.8	24.45	0.0000
Residue	1.41114 × 10^6^	113	12,487.9		
Total (Corr.)	2.63233 × 10^6^	117			
**Parameter**	**Estimate**	**Standard Error**		**T Test**	***p*-Value**
Constant	3032.31	1022.98		2.9642	0.0037
Average tree height	59.1381	8.07487		7.32372	0.0000
Richness	71.6219	11.2447		6.36938	0.0000
Dominance	−7496.03	2399.16		−3.12444	0.0023
Shannon’s diversity	−1660.65	381.752		−4.35007	0.0000

**Table 5:** Results of analysis of variance and multiple regression for aboveground tree biomass as a function of structural and ecological variables for forested abandoned mine areas in Chocó. R^2^ = 59.75%, R^2^ (adjusted) = 54.2%, and the *backward* method was used to select significant variables.

	Sum of Squares	Gf	Middle Square	F Test	*p*-Value
Model	15,812.3	4	3953.08	10.77	0.0000
Residue	10,648.2	29	367.18		
Total (Corr.)	26,460.5	33			
**Parameter**	**Estimate**	**Standard Error**		**T Test**	***p*-Value**
Constant	−35.3703	18.2267		−1.94058	0.0621
Richness	8.66422	2.02823		4.27182	0.0002
Shannon	79.2255	29.0122		2.73077	0.0106
Margalef	−57.0007	17.2118		−3.31172	0.0025
Silt	0.422064	0.20531		2.05574	0.0489

## Data Availability

The data is available in Supplementary Materials.

## Supplementary Materials

The following supporting information can be downloaded at: https://www.mdpi.com/article/10.3390/life15010098/s1, Table S1: Analysis of Variance for Aboveground biomass.
