# Supplementary material for: Influence of Environmental Factors on the Aboveground Biomass of Mature and Postmining Forests in Chocó
Source: Life (Basel). 2025 Jan 15;15(1):98. doi: 10.3390/life15010098 (PMC11766725; doi:10.3390/life15010098)
Supplement: Supplementary file 1 [file life-15-00098-s001.zip › life-3355702-supplementary.pdf]

Table S1. Analysis of Variance for Aboveground biomass _ton_ha_

Source	*Sum of Squares*	*Df*	*Mean Square*	*F-Ratio*	*P-Value*
Model	1,37551E6	32	42984,8	2,92	0,0000
Residual	1,7532E6	119	14732,8		
Total (Corr.)	3,12871E6	151			

Type III Sums of Squares

**Source**	**Sum of Squares**	**Df**	**Mean Square**	**F-Ratio**	**P-Value**
Tipe of Ecosystem	280,114	1	280,114	0,02	0,8906
Abundance	12,0478	1	12,0478	0,21	0,6532
Aluminum	15736,2	1	15736,2	1,07	0,3035
Calcium	19,7647	1	19,7647	0,00	0,9708
Clay	8387,38	1	8387,38	0,57	0,4520
Dominance	28723,8	1	28723,8	1,95	0,1652
Magnesium	3438,59	1	3438,59	0,23	0,6299
Nitrogen	39035,2	1	39035,2	2,65	0,1062
Organic matter	20830,0	1	20830,0	1,41	0,2368
pH	909,779	1	909,779	0,06	0,8042
Phosphorus	23511,4	1	23511,4	1,60	0,2090
Potassium	5046,33	1	5046,33	0,34	0,5595
Richness	5,25004	1	5,25004	0,00	0,9850
Sand	3567,84	1	3567,84	0,24	0,6235
Shannon	12790,2	1	12790,2	0,87	0,3534
Sucessional stage _years_	4372,02	1	4372,02	0,30	0,5869
Tipe of Ecosystem*Abundance	15,9215	1	15,9215	0,00	0,9738
Sucessional stage _years_*Aluminum	27770,9	1	27770,9	1,88	0,1724
Sucessional stage _years_*Nitrogen	62843,4	1	62843,4	4,27	0,0411
Sucessional stage _years_*Organic matter	87900,0	1	87900,0	5,97	0,0161
Sucessional stage _years_*Phosphorus	51291,0	1	51291,0	3,48	0,0645
Sucessional stage _years_*Richness*Organic matter	68580,2	1	68580,2	4,65	0,0330
Sucessional stage _years_*pH	6332,96	1	6332,96	0,43	0,5133
Sucessional stage _years_*Potassium	1432,46	1	1432,46	0,10	0,7557
Sucessional stage _years_*Richness	333,053	1	333,053	0,02	0,8807
Sucessional stage _years_*Shannon	45299,0	1	45299,0	3,07	0,0821
Sucessional stage _years_*Sand	12516,0	1	12516,0	0,85	0,3585
Sucessional stage _years_*Richness*Dominance	76806,5	1	76806,5	5,21	0,0242
Aluminum*Clay	16635,0	1	16635,0	1,13	0,2901
Dominance*Nitrogen	26835,8	1	26835,8	1,82	0,1797
Richness*Dominance	1856,72	1	1856,72	0,13	0,7232
Shannon*Nitrogen	52444,2	1	52444,2	3,56	0,0616
Residual	1,7532E6	119	14732,8		
Total (corrected)	3,12871E6	151			

R-Squared = 43,9642 percent
R-Squared (adjusted for d.f.) = 28,8958 percent
Standard Error of Est. = 121,379
Mean absolute error = 67,5293
Durbin-Watson statistic = 1,96236 (P=0,4087)
